# Generative Large Language Models for Psychiatric Concept Detection in Spanish-Language Electronic Health Records: Development and Evaluation Study

**DOI:** 10.2196/93328

**Published:** 2026-10-26

**Authors:** Clara Frydman-Gani, Alejandro Arias, Maria Perez-Vallejo, John Daniel Londoño Martínez, Johanna Valencia-Echeverry, Mauricio Castano Ramírez, Alex A T Bui, Nelson B Freimer, Carlos Lopez-Jaramillo, Loes M Olde Loohuis

**Affiliations:** 1 Center for Neurobehavioral Genetics, Semel Institute for Neuroscience and Human Behavior David Geffen School of Medicine University of California, Los Angeles Los Angeles, CA United States; 2 Research Group in Psychiatry (GIPSI), Department of Psychiatry School of Medicine Universidad de Antioquia Medellin, Antioquia Colombia; 3 Department of Mental Health and Human Behavior University of Caldas Manizales, Caldas Colombia; 4 Department of Radiological Sciences University of California, Los Angeles Los Angeles, CA United States

**Keywords:** LLMs, large language models, NLP, natural language processing, EHR, electronic health record, psychiatry, Spanish, concept extraction, concept detection, clinical informatics, synthetic data, generative AI

## Abstract

**Background:**

The accurate detection of clinical symptoms in electronic health records (EHRs) is pivotal for advancing large-scale genetic and longitudinal studies in psychiatry. Free-text clinical notes are an essential source of symptom-level information, particularly in psychiatry; however, the automated extraction of symptoms from clinical text remains challenging.

**Objective:**

We aimed to evaluate the ability of open-source generative large language models (LLMs) to detect psychiatric symptom-level concepts from clinical text in Spanish and to develop a new fine-tuned, open-source LLM that can be shared without risk of exposing patient information.

**Methods:**

We tested 11 open-source generative LLMs for their ability to detect 109 psychiatric concepts from clinical text, using annotated EHR notes from a psychiatric hospital in Colombia. The LLMs were evaluated both “out-of-the-box” and after fine-tuning and compared against a traditional natural language processing (tNLP) method developed from the same data. To develop a fine-tuned LLM that can be shared without risk of exposing patient information, we created a novel synthetic dataset, leveraging real concept annotations while generating fully synthetic clinical text, and fine-tuned a top-performing LLM on these data. Fine-tuned model generalizability was assessed through cross-site evaluation using data from a second independent hospital in Colombia.

**Results:**

We show that while base LLM performance was poor to moderate (zero-shot: 0.2-0.6 macro-*F*_1_; few-shot: 0.2-0.74 macro-*F*_1_), it improved significantly after fine-tuning (0.75-0.86 macro-*F*_1_), with several fine-tuned LLMs outperforming the tNLP method. In total, 100 psychiatric concepts could be reliably detected (*F*_1_>0.8) using either a fine-tuned LLM or tNLP. Using the synthetic dataset, we developed Mistral-small-psych, a new LLM capable of detecting psychiatric concepts from Spanish clinical text with performance comparable to models trained on real EHR data (macro-*F*_1_=0.79) while remaining fully distributable. In cross-site evaluations, most LLMs generalized well, with small out-of-domain performance losses (macro-*F*_1_ decrease of 0.02-0.16).

**Conclusions:**

This work provides the first large-scale, cross-institutional evaluation of state-of-the-art LLMs for psychiatric concept detection in Spanish EHRs, introduces a privacy-preserving pathway for building shareable clinical natural language processing models, and presents a new open model for extracting symptom-level psychiatric information from Spanish clinical text.

## Introduction

The large-scale extraction of psychiatric symptom and concept mentions from electronic health records (EHRs) enables epidemiological, translational, and genetic studies that transcend the boundaries of traditional diagnostic categories of severe mental illnesses [1-3]. Moreover, fine-grained psychiatric symptoms and social determinants of health can be used as features in machine learning models for predicting clinical outcomes, such as diagnostic conversion, risk of relapse, suicidality, and treatment response [4-7]. Free-text notes within the EHR are a valuable source of such symptom-level information, particularly in psychiatry, where the absence of biomarkers means many relevant phenotypes (including behaviors, affect, and mental status findings) are recorded through patient self-reports and clinicians’ narrative observations rather than structured measurements. Detecting these concepts is challenging, as psychiatric language can be highly variable: the same concept may be expressed through variable language, colloquial descriptions, abbreviations, or institution-specific phrasing, and its interpretation often depends on context. These challenges make scalable psychiatric concept detection from EHR text an important and technically difficult clinical task.

To this end, natural language processing (NLP) techniques, and specifically large language models (LLMs), are an exciting, quickly evolving frontier enabling the automated and scalable extraction of psychiatric symptoms from clinical text. Generative LLMs are neural network-based NLP systems, typically built on transformer architectures [8]. Applications of such models build upon their extensive pretraining to complete a wide range of downstream tasks, and their performance for domain-specific uses can be further refined using in-context learning or fine-tuning. Generative LLMs have demonstrated impressive performance in various clinical applications [9-12].

LLMs have been applied to automate the extraction of clinical concepts, symptoms, and other health-related entities from English-language clinical text [13-15]. While some studies have begun to explore the potential of LLMs in extracting psychiatric concepts from text, current efforts have several limitations. First, existing efforts typically target a small set of features [16-19], or focus on one or few, typically encoder-based LLMs [20-22]. Second, there is limited availability of large-scale, high-quality annotated EHR data [3,23], which is essential for fine-tuning and accurate evaluation of model performance. This limitation is particularly consequential for psychiatry, where expert annotation is required to distinguish overlapping, subjective, variable, ambiguous, and context-dependent fine-grained concepts that may not correspond directly to diagnostic codes. Given this limitation, many studies rely on non-EHR text sources, such as social media and instant messaging platforms [24-26], potentially limiting their applicability to clinical settings; others use clinical data from a single source [27] and are unable to test generalizability across settings. Third, the application of closed-source generative LLMs on EHR-derived text may raise concerns around patient privacy and potential leakage of protected health information [23]. This constraint has motivated the use of open-source LLMs [28,29], which, while applicable to EHR data, cannot be publicly released after fine-tuning due to the risk of leaking patient data. Fourth, while recent work has begun to explore the utility of NLP for targeting certain clinical [30,31] and psychiatric [32] concepts in Spanish-language text, the capabilities of generative LLMs to extract psychiatric concepts from Spanish clinical texts remain understudied. This represents a critical gap, given that Spanish is the world’s second most common native language, and is widely used in clinical practice across multiple countries. Nevertheless, the development of clinical NLP tools that could serve large Spanish-speaking populations remains limited.

To address these gaps, we evaluated the performance of 11 state-of-the-art open-source generative LLMs for the document-level extraction of 136 psychiatric concepts from Spanish-language EHR documents derived from psychiatric hospitals in Colombia, including symptoms related to mood and affect, substance use, perception, and behavior. Models were applied using both zero- and few-shot prompting, with the latter providing a small number of examples taken from annotated documents during inference. Next, we fine-tuned all LLMs using a clinician-annotated corpus of EHR documents and compared their performance to that of a pattern-based traditional natural language processing (tNLP) method previously developed in the same setting [33]. We then developed Mistral-small-psych, a new open-source lightweight generative LLM that circumvents the data leakage limitation of LLMs by being trained on synthetic data generated using real psychiatric symptom annotations. Finally, we evaluated all fine-tuned models on 2 EHR-derived test sets to assess performance and generalizability across hospital settings.

## Methods

### Models, Settings, and Data

#### Model Selection

We selected an array of widely-used generative LLMs of varying sizes for benchmarking and fine-tuning. Two key criteria in model selection were pretraining on corpora that included Spanish-language text and open-source availability. The latter requirement was necessary, given that the evaluation and fine-tuning data were derived from EHRs and had to be contained in a secure environment, requiring the ability to deploy and run all models locally.

The following models were selected for this study: Llama3-8B-Instruct (hereafter: “Llama3-8B”) and Llama3-70B-Instruct (“Llama3-70B”) released by Meta AI [34]; Qwen2.5-7B-Instruct (“Qwen2.5-7B”), Qwen2.5-14B-Instruct (“Qwen2.5-14B”), and Qwen2.5-32B-Instruct (“Qwen2.5-32B”) by Alibaba Cloud [35]; Mistral-7B-Instruct-v0.3 (“Mistral-7B”), Mistral-small-24B-Instruct-2501 (“Mistral-small”) and Mixtral-8x7B-Instruct-v0.1 (“Mixtral-8x7B”) by Mistral AI [36,37]; Mistral-nemo-Instruct-2407 (“Mistral-nemo”) by Mistral AI and NVIDIA [38]; and Aya-23-8B (“Aya-8B”) and Aya-23-35B (“Aya-35B”) by Cohere [39]. An overview of the selected LLMs is shown in Table 1.

**Table 1:** Generative large language models evaluated for psychiatric concept detection from Spanish electronic health record notes from Clínica San Juan de Dios Manizales and Hospital Mental de Antioquia in Colombia.

Full model name	Size (parameters)	Developer	License
Meta-Llama-3-8B-Instruct	8B	Meta	Llama3
Meta-Llama-3-70B-Instruct	70B	Meta	Llama3
Qwen2.5-7B-Instruct	7B	Alibaba Cloud	Apache license 2.0
Qwen2.5-14B-Instruct	14B	Alibaba Cloud	Apache license 2.0
Qwen2.5-32B-Instruct	32B	Alibaba Cloud	Apache license 2.0
Aya-23-8B	8B	Cohere Labs and Cohere	CC-BY-NC
Aya-23-35B	35B	Cohere Labs and Cohere	CC-BY-NC
Mistral-7B-Instruct-v0.3	7B	Mistral AI	Apache license 2.0
Mistral-Nemo-Instruct-2407	12B	Mistral AI and NVIDIA	Apache license 2.0
Mistral-Small-24B-Instruct-2501	24B	Mistral AI	Apache license 2.0
Mixtral-8x7B-Instruct-v0.1	8x7B	Mistral AI	Apache license 2.0

#### Setting

We used data from 2 distinct settings in the model evaluation and fine-tuning processes: the first, Clínica San Juan de Dios Manizales (CSJDM) in Manizales, Colombia, serves a population of ~1 million individuals in the department of Caldas. The second, Hospital Mental de Antioquia (HOMO), is a larger psychiatric institution and the only public psychiatric hospital in the department of Antioquia, Colombia. Data from CSJDM were used to evaluate and fine-tune all LLMs, as well as for the construction of a synthetic dataset, while data from HOMO were subsequently used as an out-of-domain test set to evaluate the generalizability of all fine-tuned models.

#### Real EHR Data and Concept Annotation

We used a previously curated corpus of 2000 clinical documents pertaining to 1821 patients from the CSJDM clinical notes [33]. Documents were selected, annotated, and split into training and test sets as described by De La Hoz et al [33] (Table 2). Briefly, documents were selected from intake, emergency department, and outpatient notes, prioritizing concept-rich fields. Then, 2 expert clinicians—a psychiatrist and a clinical psychologist—annotated the documents for 136 symptom-level concepts, including concepts relating to mood or affect (such as “guilt,” “hopelessness,” and “depressed mood”), thought and cognition (“delusions,” “circumstantiality,” and “suicidal ideation”), and behavior and motor function (“aggression,” and “disorganized behavior”), among others (Figure 1A and Table S1 in Multimedia Appendix 1 for a list of all concepts). Documents were split into training and test sets using a concept-stratified procedure designed to ensure adequate representation of each concept in both partitions. Since applying a random 80%-20% split could have underrepresented low-frequency concepts in the test set, we selected test documents in an iterative process such that at least 20% of each eligible concept’s annotated instances appeared in the test set, while at least 50% remained in the development set. This procedure resulted in a CSJDM development/test split of 1642/358 documents, corresponding to approximately 82%/18% of the annotated corpus. Interannotator agreement on the training set was assessed using Cohen κ [40]. Test set documents (n=358) underwent a joint revision process resolving any discrepancies among the annotators to ensure a rigorous reliability standard for evaluation. For the fine-tuning step, the initial CSJDM training set (n=1642) was split into training (n=1477) and validation (n=165) sets. The same process—including annotation by the same team of clinicians and a discrepancy resolution step—was applied to HOMO EHR documents to generate the HOMO test set, consisting of 309 documents (Table 2). HOMO documents were intentionally reserved for external cross-site evaluation and were not used for model training or hyperparameter selection, because training on both institutions would preclude assessment of transportability from CSJDM to an independent hospital setting.

**Table 2:** Descriptions of the annotated Spanish electronic health record document corpora used for psychiatric concept extraction at Clínica San Juan de Dios Manizales (CSJDM) and Hospital Mental de Antioquia (HOMO) in Colombia, including training and test set partitions, note sections included, and number of unique patients represented in each hospital corpus^a^.

	CSJDM	HOMO
Annotated documents	2000	2000
Development set documents	1642	Not used
Test set documents	358	309
Note sections included	Chief complaint, thought content, analysis, current illness, and objective	Chief complaint, thought content, analysis, affect, and sensory-perception
Unique patients	1821	1828

^a^Documents were annotated for 136 psychiatric concepts by expert clinicians, with discrepancy resolution applied to both test sets. The corpus from CSJDM was used for model development and internal testing, whereas the HOMO corpus was used as an external cross-site test set to evaluate generalizability of the fine-tuned models.

**Figure 1:**
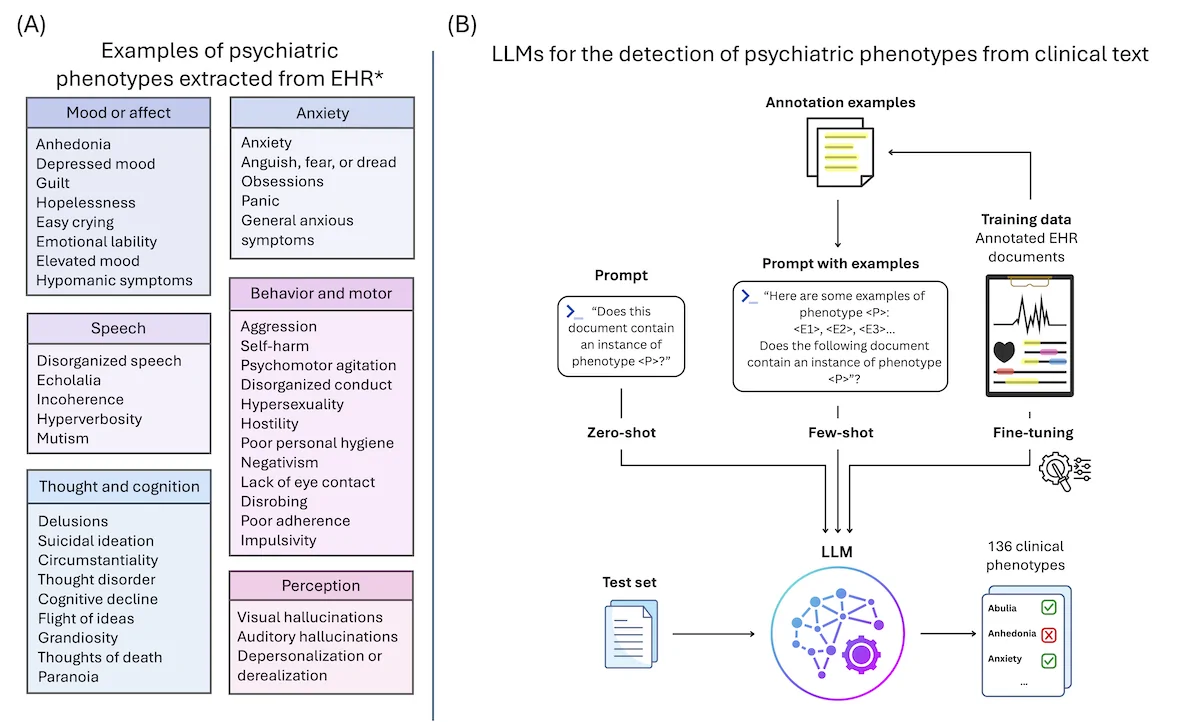
Overview of extracted psychiatric concepts and study design. (A) Examples of the psychiatric concepts extracted from Spanish EHR documents in this study, spanning domains such as mood and affect, thought and cognition, perception, and behavior and motor function. *The full list of 136 concepts is provided in Table S1 in Multimedia Appendix 1. (B) Overview of the LLM fine-tuning and evaluation workflow. In total, 11 open-source generative LLMs were evaluated for the detection of 136 psychiatric concepts from Spanish EHR documents from Colombia. Models were evaluated using zero-shot prompting (providing no additional information aside from the prompt), few-shot prompting (providing 5 annotated examples of the target concept as part of the prompt), and fine-tuning on a training set of clinician-annotated documents from Clínica San Juan de Dios Manizales via instruction-tuning. All models were evaluated on an internal test set from Clínica San Juan de Dios Manizales, and fine-tuned models were also evaluated on an external test set from Hospital Mental de Antioquia. EHR: electronic health record; LLM: large language model.

#### Synthetic Data

To enable model fine-tuning and publication without risk of protected health data leakage, we created a synthetic dataset of documents not based on real clinical text. To do so, we prompted Llama3-70B to generate sentences emulating clinical notes written by a clinician, containing real CSJDM EHR training set annotations (Note S1 in Multimedia Appendix 2). To ensure data privacy, the LLM was prompted using only the specific annotated spans—previously published brief phrases, manually reviewed to ensure no identifiable information was included. As such, the model was at no point exposed to patient information, and the generated sentences carried no risk of containing it. Additionally, seeing that the real CSJDM data included many sentences with mostly somatic information and no mention of psychiatric symptoms (such as laboratory test results and depiction of somatic symptoms), we also prompted Llama3-70B to generate clinical sentences containing demographic and somatic clinical information rather than psychiatric symptoms (hence referred to as “somatic or other sentences”; Note S1 in Multimedia Appendix 2). Synthetic sentence generation was conducted, setting the generation temperature parameter of Llama3-70B to 0.8 for the sentences containing the annotations, and 0.99 for the somatic or other sentences, to promote sentence diversity, given multiple iterations of identical prompts. The synthetic generation prompts were designed to produce clinical-style sentences containing the target symptom mentions, but did not explicitly sample assertion-status variants such as negated, historical, hypothetical, uncertain, or nonpatient mentions.

Synthetic sentences underwent a quality control step, ensuring the inclusion of the intended annotations (Note S1 in Multimedia Appendix 2). Then, we sampled 3440 sentences while prioritizing diverse annotation examples, as well as 300 random somatic or other sentences, for a total of 3740 synthetic sentences (Note S1 in Multimedia Appendix 2). Finally, sentences were shuffled and assembled into groups of 1-6 sentences to produce longer “documents.”

As the LLM was prompted to generate synthetic sentences intended to emulate clinicians’ clinical notes, the synthetic data often contained phrases depicting additional mentions of various psychiatric symptoms, other than the one the LLM was explicitly prompted for. To recover and label these additional symptoms incidentally appearing in the synthetic dataset and create more accurately labeled training data, we applied the tNLP method to the synthetic documents as a weak-labeling approach. The final synthetic training set contained annotations for both the originally targeted concepts and those identified by the tNLP method.

### Inference Applications

#### Zero-Shot Prompting

The extraction task was defined as explicit psychiatric concept mention detection rather than assertion status–resolved patient-level phenotyping. All LLMs were evaluated on the CSJDM test set, prompted to provide a binary response indicating the presence of a given concept in each test set document (Figure 1B and Note S2 in Multimedia Appendix 2 for full detail). For this evaluation, we used zero-shot prompting (ie, prompting the models to extract psychiatric symptoms providing no examples or additional information, with the full prompt included in Note S2 in Multimedia Appendix 2). LLMs were prompted separately for each document-concept pair, with no shared context between prompts, ensuring that no information was carried over across queries. This design enabled standardized concept-specific scoring against the reference annotations and avoided requiring models to select from the full ontology of more than 100 concepts in a single prompt. All evaluation runs were conducted with the temperature parameter set to 0 to control sampling randomness and promote deterministic and reproducible outputs. All LLMs were served via Ollama (version 0.3.14), an open-source framework for the local deployment of LLMs. Models were loaded in 4-bit quantization on instances using 1 or 4 NVIDIA A10G Tensor Core graphics processing units (GPUs), with 24 or 96 GB of video random-access memory (VRAM), respectively. The number of GPUs used for each model was selected according to model size and memory requirements, with larger models requiring 4 A10G GPUs.

#### Few-Shot Prompting

Few-shot prompting consists of supplying a small number of examples of the task (typically ranging from 2 to 10) as part of the prompt. This approach leverages in-context learning, wherein the model conditions its response on the examples and information provided when prompting, with no modification to its underlying weights; it has been demonstrated to improve model performance, in some cases, yielding comparable results to those of model fine-tuning, but at a fraction of the necessary labeled data and computational cost [41].

For each concept, we selected up to 5 examples of annotated spans from the CSJDM training set to construct the few-shot prompts, while ensuring a balance of example diversity and representativeness (Note S3 in Multimedia Appendix 2). We first ranked all annotated instances by frequency in the training set, then randomly sampled 5 distinct examples from the 10 most frequent distinct annotations. When fewer than 10 or 5 unique annotations were available for the concept, we used all available instances for random sampling, or all distinct annotations available, to construct the few-shot prompt, respectively (Figure 1B and Note S3 in Multimedia Appendix 2). As before, each combination of test set document-concept was prompted separately, with no context carried between prompts, and the temperature parameter set to 0 to promote reproducibility. For each concept, the selected few-shot examples were held fixed and reused for every model and every test document, ensuring that differences in few-shot performance were not attributable to differences in the examples provided.

### Fine-Tuning

#### Fine-Tuning on CSJDM Data

We used the CSJDM training set to fine-tune all LLMs (Figure 1B). The number of training epochs was selected independently for each LLM from a shared hyperparameter space, reflecting model-specific convergence behavior. LLMs were trained for 1-4 epochs on G5 Amazon Web Services (AWS) instances with 1 (Llama3-8B, Mistral-7B, Mistral-nemo, Mistral-small, Qwen2.5-7B, Qwen2.5-14B, Qwen2.5-32B, and Aya-8B) or 4 (Aya-35B, Mixtral-8x7B, and Llama3-70B) NVIDIA A10G Tensor Core GPUs, with 24 GB of VRAM each. To optimize memory use and training time, we used Unsloth [42] in the fine-tuning of Llama3-8B, Mistral-7B, Mistral-nemo, Mistral-small, Qwen2.5-7B, Qwen2.5-14B, and Qwen2.5-32B models, and Axolotl for all other models. To further reduce memory and training time requirements, all models were downloaded in 4-bit quantization and fine-tuned using low-rank adaptation (LoRA), a parameter-efficient fine-tuning method in which the original model’s weights remain frozen, while smaller rank decomposition matrices injected into the model’s layers are trained during fine-tuning [43]. LoRA is a widely used method in LLM fine-tuning, enabling a significant reduction in the number of trainable parameters without significant losses in performance. Fine-tuning was performed using a multilabel document-level extraction format [14], where each training example consisted of 1 clinical document, and the target output contained the list of detected concepts in that document. To address the limitations of LLM output explainability, we used an instruction-tuning approach in which the models were trained to produce not only the concepts detected in the text but also their corresponding labeled spans. Importantly, fine-tuned models were also trained using annotated context cues, enabling downstream postprocessing or future modeling of assertion status, including negation, temporality, family history, and uncertainty. Models were instructed to extract concepts using free-text generation rather than ontology-constrained decoding, and the generated outputs were subsequently mapped to the predefined concept ontology during evaluation. Additional information on the fine-tuning process can be found in Note S4 in Multimedia Appendix 2.

#### Fine-Tuning on Synthetic Data

The top-performing base model from the real-data fine-tuning step—prioritizing smaller model size in cases of similar performance to enable greater accessibility—was fine-tuned using the synthetic dataset. The selected base model was downloaded in 4-bit quantization and fine-tuned on the synthetic dataset using Unsloth and LoRA. As in the real-data fine-tuning, we used an instruction-tuning format in which the target output included both the detected concepts and their corresponding labeled spans (Note S5 in Multimedia Appendix 2). Performance was evaluated after fine-tuning both on the original synthetic sentences (without the tNLP weak labeling) and the synthetic training data after the tNLP labeling augmentation. Because the synthetic corpus was augmented using weak labels generated by the tNLP method, we did not treat the fine-tuned LLM as an independent comparator to the tNLP, and therefore do not present a head-to-head performance comparison between these methods.

### Evaluation

#### Response Parsing

For zero-shot and few-shot evaluations, nonbinary responses were classified as negative, as manual review of these responses indicated that these were rare and primarily reflected safety-oriented refusals to engage with the instruction, general descriptions of the note’s main themes with cautionary guidance, or explanatory negative answers rather than correct positive assertions.

#### Model Performance Metrics

Performance at all levels (for both inference and fine-tuned models) was evaluated on the CSJDM test set against the clinicians’ annotations, using precision, recall, macro-*F*_1_, micro-*F*_1_, and balanced accuracy (Note S6 in Multimedia Appendix 2).

To ensure a minimum level of reliability in performance metrics, we report performance only for concepts with at least 3 instances in the CSJDM training and test sets (N concepts=109).

For interpretation, overall model performance based on macro-*F*_1_-scores was considered poor (≤0.6), moderate (0.6-0.75), good (0.75-0.9), or excellent (≥0.9).

#### Comparison to tNLP Method

LLM performance (both for inference and after fine-tuning) was benchmarked against a tNLP method previously developed from the same CSJDM training data [33] and evaluated on the CSJDM test set. Performance comparisons of this method with the LLMs are based on the 88 concepts evaluated using tNLP.

#### Cross-Hospital Transportability Evaluation

All fine-tuned models underwent subsequent evaluation on the HOMO out-of-domain test set to assess their performance in external settings.

### Statistical Analysis

Model performance was evaluated separately for each concept, and aggregated performance summaries (eg, mean recall, macro-*F*_1_, and micro-*F*_1_) are reported to facilitate general large-scale comparisons across models. Given that all experiments were conducted with the temperature hyperparameter set to 0 to promote deterministic outputs and reproducibility, measures of variability reported in this study reflect concept-level performance dispersity rather than uncertainty due to stochastic sampling.

For comparisons between LLMs and the rule-based tNLP method, we used a Wilcoxon signed rank test on per-concept *F*_1_-scores, with false discovery rate correction applied to account for multiple comparisons with α=.05. Correlation analyses between model performance and concept characteristics (eg, training frequency, interannotator agreement, and lexical variability) were conducted using Spearman rank correlation analyses. All statistical analyses were performed using Python (version 3.10; Python Software Foundation).

### Synthetic Training Set Size Sensitivity Analysis

To assess how model performance changed as a function of the synthetic training dataset size, we created multiple training subsets by randomly sampling 3 subsets each of 250, 500, 750, and 1000 synthetic documents from the original synthetic training data. Three independent subsets per size were drawn to reduce the chance that results at a given subset size were driven by a single unusually easy or difficult random sample, providing a practical estimate of sampling variability while limiting the computational cost of repeated LLM fine-tuning. Separate models were fine-tuned on each dataset, and their performance was evaluated on the CSJDM test set. To assess whether random subsampling substantially altered concept prevalence distributions, we additionally compared concept-level prevalence vectors between each subset and the full synthetic training set using Spearman correlation, Jensen-Shannon divergence, and Hellinger distance. Spearman correlation was used to assess preservation of concept frequency rankings across subsets, whereas Jensen-Shannon divergence and Hellinger distance were used to quantify distributional similarity and prevalence drift between concept prevalence vectors.

### Inference Runtime, Electricity Consumption, and Cost

The application of generative LLMs for inference typically requires the use of specialized hardware (ie, GPUs). These applications could incur substantial costs, time, and carbon footprint when applied to real-world EHR repositories, which can consist of millions of documents. To benchmark these expenses, we report the average runtime (in seconds), system electricity consumption (in watt-hours), and cost (in cents) for zero-shot inference with each LLM via Ollama (version 0.3.14) on a single concept across 100 documents. All benchmark runs were standardized on an AWS g5.12xlarge EC2 instance in the US West (Oregon) Region, equipped with 4 NVIDIA A10G Tensor Core GPUs with 24 GB of VRAM each, for a total of 96 GB of GPU VRAM. Consumption measurements were performed using CodeCarbon, a software tool for tracking energy use and estimating carbon emissions from computational workloads [44]. Runtime measurements included writing generated outputs to file but excluded model loading time and JSON parsing, because the objective was to compare generation-time resource requirements rather than implementation-dependent preprocessing or postprocessing steps. Costs were estimated from runtime using AWS g5.12xlarge on-demand pricing for the US West (Oregon) Region.

### Ethical Considerations

This study was conducted in compliance with applicable Colombian and United States regulations and institutional guidelines. The study procedures were reviewed and approved by the University of California, Los Angeles Medical Institutional Review Board 3 (IRB#20-000149, IRB#20-001537, and IRB#16-002084), the Comité de Ética del Instituto de Investigaciones Médicas at Universidad de Antioquia, the Comité de Ética en Investigación de la E.S.E. Hospital Mental de Antioquia, and the Comité de Bioética de Clínica San Juan de Dios Manizales. Participants provided signed informed consent for participation in the studies approved under IRB#20-000149, IRB#20-001537, and IRB#16-002084. Access to EHR data from CSJDM was approved by the Comité de Bioética de Clínica San Juan de Dios Manizales with a waiver of informed consent for secondary use of clinical data. For data obtained from HOMO, all participants provided informed consent authorizing access to and secondary analysis of their EHR data. All secondary analyses reported in this study were conducted under the ethics approvals and consent provisions described earlier. To protect participant privacy and confidentiality, all analyses were conducted using deidentified study data stored and processed in secure, access-controlled computing environments. Model development and evaluation were performed on clinical text within these protected environments. Publicly released materials do not include identifiable patient information. The synthetic dataset was generated from previously published, manually reviewed concept annotation spans that did not contain protected health information, and the released synthetic text contains no patient identifiers. No images or supplementary materials contain identifiable individual participants. No identification of individual participants is possible from images or results of the paper or multimedia appendices. Finally, participants were not compensated for the analyses described in this paper.

## Results

### Model Performance

#### Zero-Shot Prompting

All LLMs were assessed for their capabilities to detect 109 psychiatric symptom-level concepts associated with mood and affect, perception, thought and cognition, and behavior, among other domains (Figure 1 and Table S1 in Multimedia Appendix 1). Overall, model performance was poor to moderate in zero-shot evaluations across the 109 concepts tested (Figure 2 and Table 3). While larger models within the same family often performed better than smaller ones, this trend did not extend across different model families (Figure S1A in Multimedia Appendix 2).

**Figure 2:**
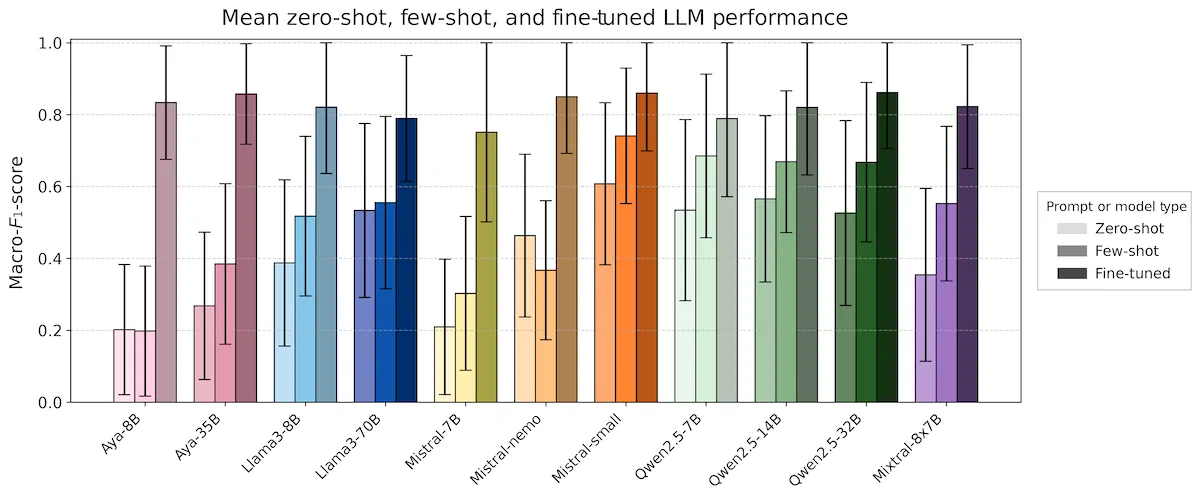
Macro-*F*_1_ performance of zero-shot, few-shot, and fine-tuned generative LLMs for psychiatric symptom-level concept extraction in Spanish electronic health record documents. Mean concept-level *F*_1_-scores (macro-F1s) are shown for each model evaluated on the Clínica San Juan de Dios Manizales test set (n=358 documents) across 109 psychiatric concepts with at least 3 instances in both the training and test sets. Grouped bars for each of the 11 base models represent zero-shot prompting (left), few-shot prompting with up to 5 annotated examples of the target concept (middle), and performance after fine-tuning on a training set of clinician-annotated electronic health record documents from the same hospital (right). Error bars indicate 1 SD in *F*_1_-scores across evaluated concepts. LLM: large language model.

**Table 3:** Performance of zero-shot (no additional information in the prompt, left), few-shot (providing 5 annotated examples of the target concept within the prompt, center), and fine-tuned (right) generative large language models for psychiatric concept extraction from Spanish electronic health record documents from Clínica San Juan de Dios Manizales in Colombia^a^.

Model	Zero-shot					Few-shot					Fine-tuned			
	Precision, mean (SD)	Recall, mean (SD)	Macro-*F*_1_, mean (SD)	Micro-*F*_1_	Precision, mean (SD)		Recall, mean (SD)	Macro-*F*_1_, mean (SD)	Micro-*F*_1_	Precision, mean (SD)		Recall, mean (SD)	Macro-*F*_1_, mean (SD)	Micro-*F*_1_
Llama3-70B	0.45 (0.27)	0.82 (0.22)	0.53 (0.24)	0.58	0.44 (0.26)		0.94 (0.09)	0.56 (0.24)	0.60	0.9 (0.14)		0.74 (0.21)	0.79 (0.18)	0.81
Llama3-8B	0.32 (0.25)	0.73 (0.27)	0.39 (0.23)	0.45	0.42 (0.24)		0.85 (0.18)	0.52 (0.22)	0.60	0.9 (0.17)		0.79 (0.22)	0.82 (0.18)	0.84
Qwen2.5-14B	0.49 (0.26)	0.82 (0.21)	0.57 (0.23)	0.59	0.58 (0.24)		0.89 (0.15)	0.67 (0.20)	0.72	0.90 (0.15)		0.79 (0.23)	0.82 (0.19)	0.84
Qwen2.5-32B	0.44 (0.27)	0.86 (0.19)	0.53 (0.26)	0.53	0.57 (0.26)		0.93 (0.11)	0.67 (0.22)	0.70	0.89 (0.14)		0.86 (0.18)	0.86 (0.16)	0.87
Qwen2.5-7B	0.56 (0.29)	0.64 (0.3)	0.53 (0.25)	0.62	0.71 (0.25)		0.72 (0.26)	0.69 (0.23)	0.74	0.88 (0.20)		0.74 (0.24)	0.79 (0.22)	0.81
Aya-35B	0.18 (0.17)	0.85 (0.15)	0.27 (0.2)	0.30	0.28 (0.21)		0.92 (0.13)	0.38 (0.22)	0.43	0.90 (0.14)		0.84 (0.17)	0.86 (0.14)	0.87
Aya-8B	0.13 (0.14)	0.89 (0.13)	0.20 (0.18)	0.24	0.12 (0.14)		0.98 (0.05)	0.20 (0.18)	0.23	0.88 (0.15)		0.82 (0.19)	0.83 (0.16)	0.84
Mixtral-8x7B	0.30 (0.26)	0.65 (0.26)	0.35 (0.24)	0.42	0.47 (0.26)		0.82 (0.18)	0.55 (0.22)	0.60	0.88 (0.16)		0.8 (0.20)	0.82 (0.17)	0.84
Mistral-7B	0.14 (0.15)	0.88 (0.17)	0.21 (0.19)	0.25	0.21 (0.19)		0.81 (0.20)	0.30 (0.21)	0.37	0.81 (0.25)		0.73 (0.27)	0.75 (0.25)	0.77
Mistral-nemo	0.49 (0.27)	0.53 (0.26)	0.46 (0.23)	0.56	0.26 (0.18)		0.88 (0.14)	0.37 (0.19)	0.45	0.90 (0.14)		0.83 (0.19)	0.85 (0.16)	0.86
Mistral-small	0.57 (0.27)	0.76 (0.24)	0.60 (0.23)	0.64	0.72 (0.24)		0.84 (0.17)	0.74 (0.19)	0.78	0.90 (0.15)		0.85 (0.20)	0.86 (0.16)	0.88
Mistral-small-psych	—^b^	—	—	—	—		—	—	—	0.82 (0.17)		0.80 (0.18)	0.79 (0.15)	0.85

^a^Mean precision, recall, *F*_1_-score (macro-*F*_1_), and micro-*F*_1_ were evaluated for 11 open-source generative large language models on the Clínica San Juan de Dios Manizales test set (n=358 documents). Models were evaluated using zero- and few-shot prompting, and after fine-tuning on a training set of clinician-annotated electronic health record documents from Clínica San Juan de Dios Manizales (n=1477 documents, right). Values are reported as mean (SD) across all evaluated psychiatric concepts with at least 3 instances in both the training and test sets (n=109 concepts). Mistral-small-psych (bottom) is a fine-tuned version of Mistral-small tuned on synthetic data only. Micro-*F*_1_ is a single aggregate score calculated by pooling true positives, false positives, and false negatives across all evaluated phenotypes and therefore is not accompanied by an across-phenotype SD.

^b^Not applicable.

Mistral-small (macro-*F*_1_=0.6, micro-*F*_1_=0.64) and Qwen2.5-14B (macro-*F*_1_=0.57, micro-*F*_1_=0.59) achieved the best overall zero-shot performance. However, concept-level performance varied substantially: Mistral-small and Qwen2.5-14B achieved strong performance (*F*_1_≥0.8) on 25 and 21 concepts, respectively, but showed poor *F*_1_ performance (≤0.6) on 57 and 65 concepts, reflecting the variability in the models’ capability of detecting specific concepts (Table S2 in Multimedia Appendix 1 and Figure S2A in Multimedia Appendix 2). While most models had strong (≥0.8) recall, they were limited by their precision. In particular, the smaller Mistral-7B and Aya-8B showed good recall (0.88 and 0.89, respectively) but markedly low precision (0.14 and 0.13, respectively), suggesting a tendency toward positive labeling with limited discriminative ability in this setting. The tNLP method significantly outperformed all LLMs (Table S5 in Multimedia Appendix 1 and Figure S3 in Multimedia Appendix 2), achieving a macro-*F*_1_-score of 0.85, with particularly notable advantages in some concepts, including “negativism” and “thought disorder” (Table S6 in Multimedia Appendix 1 and Figure S2B in Multimedia Appendix 2). In contrast, a small number of concepts, including “weight loss” and “poor personal hygiene,” were better detected by several LLMs.

#### Few-Shot Prompting

Few-shot prompting improved macro-*F*_1_ for all models except Mistral-nemo (–0.09) and Aya-8B (no change; Figure 2 and Table 3), with concept-level performance shown in Figure S4 in Multimedia Appendix 2. Mistral-small achieved the highest few-shot performance (macro-*F*_1_=0.74, SD 0.19, micro-*F*_1_=0.78) following an increase in both precision and recall following the addition of examples to the prompt. The concepts with the greatest average improvement from zero- to few-shot across all LLMs were “low energy” (+0.4 *F*_1_, SD 0.22), “hyperverbosity” (+0.37 *F*_1_. SD 0.16), “psychomotor delay” (+0.39 *F*_1_, SD 0.35) and “poor insight” (+0.39 *F*_1_, SD 0.21); Table S3 in Multimedia Appendix 1). Despite this marked improvement for most models, the tNLP method significantly outperformed all LLMs in the few-shot setting (Tables S5 and S6 in Multimedia Appendix 1 and Figure S3 in Multimedia Appendix 2).

#### Real-Data Fine-Tuning

All LLMs saw dramatic improvement in performance following the fine-tuning process, displaying good performance (Figure 2, Table 3, and Table S4 in Multimedia Appendix 1). The best-performing fine-tuned models were Aya-35B, Mistral-small, and Qwen2.5-32B (macro-*F*_1_=0.86 for all). Aya-8B displayed the greatest improvement compared to the base model’s zero-shot performance, with a macro-*F*_1_ increase of +0.63 from its zero-shot macro-*F*_1_ of 0.2.

While performance was more variable—across both models and concepts—in rarer concepts, it generally improved and converged as the concepts’ training set frequency increased (Figure 3 and Figure S5A in Multimedia Appendix 2). Nevertheless, we still observed variation in some cases, both between models (eg, Qwen2.5-7B’s markedly low performance for “poor adherence”) and for specific concepts (eg, all models’ relatively poor performance for “negativism,” “adverse effects,” and “poor response to psychotropic medications”). In contrast, some concepts, like “hypothymia,” had high (*F*_1_≥0.89) performance for all models despite having relatively low frequencies in the training data.

Unlike the base models, the tNLP method did not significantly outperform any of the fine-tuned LLMs, except for Llama3-70B (adjusted *P*=.01; Table S5 in Multimedia Appendix 1). Moreover, several models—including Qwen2.5-32B, Mistral-nemo, Mistral-small, Llama3-8B, Aya-8B, and Aya-35B—achieved higher macro-*F*_1_-scores than the tNLP method (Table S6 in Multimedia Appendix 1 and Figure S3 in Multimedia Appendix 2). Still, the tNLP method outperformed all fine-tuned LLMs in 9 concepts, including “cocaine” (median Δ*F*_1_=0.28) and “TECAR” (terapia electroconvulsiva con anestesia y relajación, or electroconvulsive therapy, median Δ*F*_1_=1), both of which are captured by very few (2-3) distinct patterns (Figure S5B in Multimedia Appendix 2). In contrast, all fine-tuned LLMs outperformed the tNLP method in the detection of 10 concepts, including “change in weight” (median Δ*F*_1_=0.22) and “altered memory” (median Δ*F*_1_=0.33), and were able to successfully detect concepts for which no patterns were developed due to low frequency or interannotator agreement in the training data, including “psychomotor agitation” and “panic” [33]. Overall, 100 concepts were successfully extracted (*F*_1_>0.8) using at least 1 of the fine-tuned LLMs or the tNLP method.

**Figure 3:**
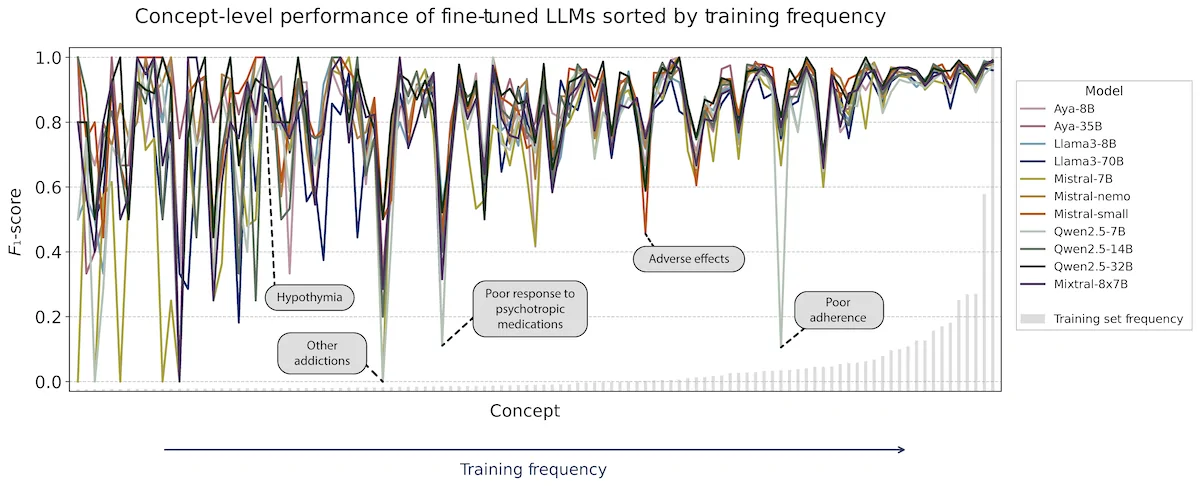
Concept-level *F*_1_ performance of 11 fine-tuned generative LLMs in relation to concept frequency in the training data. Each colored line shows per-concept *F*_1_-scores for a fine-tuned LLM evaluated on a Clínica San Juan de Dios Manizales test set (n=358 electronic health record documents). Psychiatric concepts are ordered from left to right by their frequency in the clinician-annotated training set from Clínica San Juan de Dios Manizales used for fine-tuning. Gray bars at the bottom indicate relative training set frequency of each concept. Highlighted concepts illustrate examples of performance patterns across models, including concept-specific challenges: consistently low performance for broader or more lexically variable concepts (“other addictions,” “poor response to psychotropic medications,” and “adverse effects”), model-specific challenges (“poor adherence”), and high performance despite low training-set frequency for a lexically consistent concept (“hypothymia”). LLM: large language model.

#### Synthetic-Data Fine-Tuning

The final synthetic dataset we created to develop a publishable LLM consisted of 1223 documents (mean number of tokens per document 83.72, SD 51.91). Application of the tNLP method to the synthetic data boosted the number of labeled instances from 3440 to 5427 (Figure 4A and Figure S7B in Multimedia Appendix 2). Given Mistral-small’s performance in both fine-tuning and few-shot settings along with its smaller size, we selected it as the base model to fine-tune on the synthetic dataset, creating Mistral-small-psych. Base Mistral-small was fine-tuned for 9 epochs, during approximately 4 hours on a single NVIDIA A10G GPU with 24 GB of VRAM (Note S5 in Multimedia Appendix 2).

Mistral-small-psych showed good performance overall (macro-*F*_1_=0.79, SD 0.15; micro-*F*_1_=0.85; Table 3 and Table S4 in Multimedia Appendix 1), an improvement of +0.19 from the base model’s macro-*F*_1_. Moreover, we found that augmenting the original synthetic dataset with the tNLP-generated labels led to significant gains in performance, as fine-tuning on the original synthetic training data (without the tNLP system labeling) achieved a macro-*F*_1_-score of only 0.49. Mistral-small-psych outperformed all base models in both zero- and few-shot settings (Figure S8 in Multimedia Appendix 2 and Table 3) and had comparable performance to that of LLMs fine-tuned on real CSJDM data (Figure 4B). Of note, it achieved a macro-*F*_1_-score that was equal or higher than that of 3 models fine-tuned on real EHR data: Llama3-70B, Qwen2.5-7B, and Mistral-7B. Moreover, despite being fine-tuned exclusively on synthetic data, Mistral-small-psych achieved a mean precision of 0.82 (SD 0.17; Table 3).

**Figure 4:**
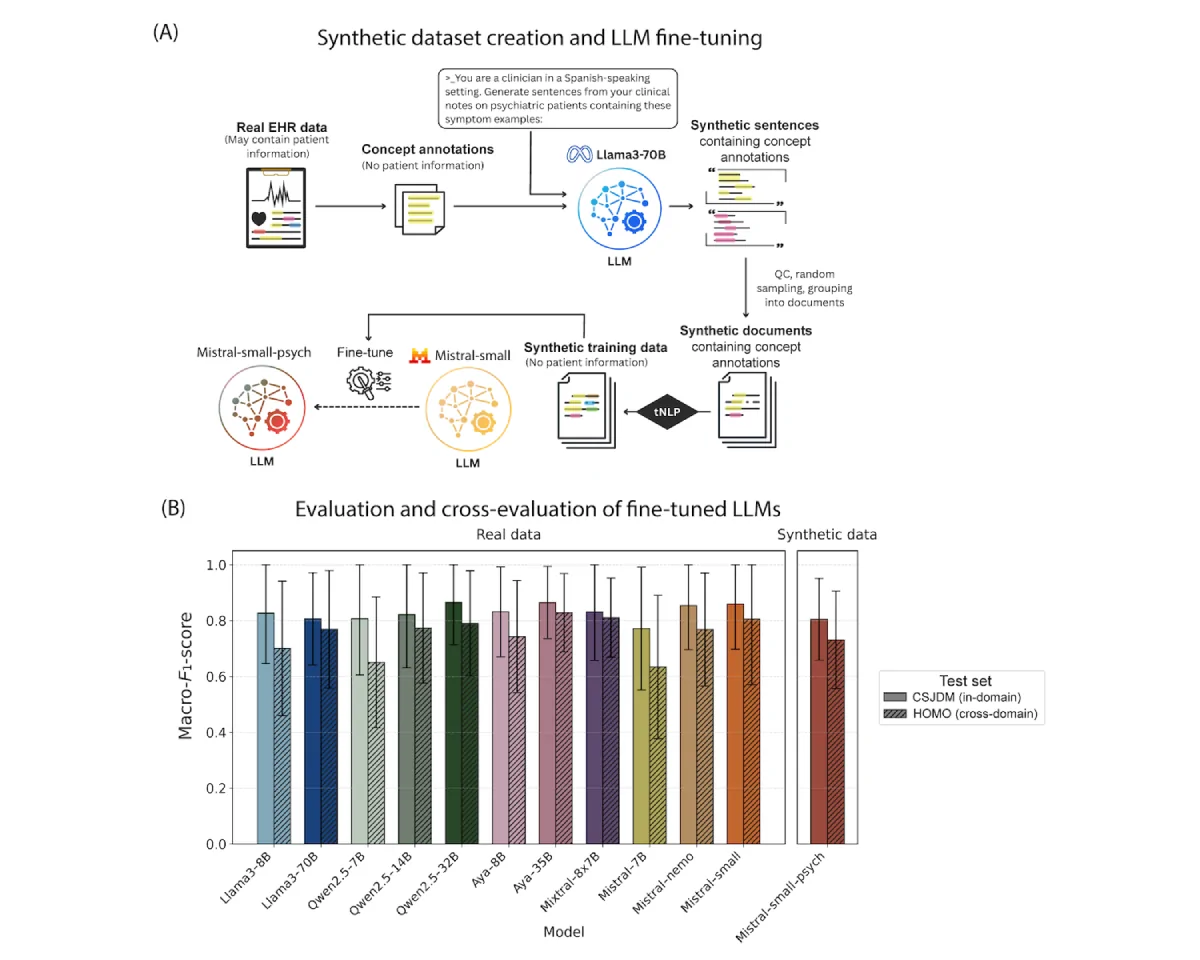
Development and evaluation of Mistral-small-psych, a generative LLM fine-tuned exclusively on synthetic data for psychiatric concept extraction from Spanish clinical text. (A) Overview of the development workflow for Mistral-small-psych: Llama3-70B was prompted to generate synthetic clinical sentences containing published concept annotation examples extracted from real clinician-annotated EHR data from Colombia. These synthetic sentences, which contained no patient information, were sampled and assembled into documents, further labeled using a tNLP. Finally, these synthetic documents were used to fine-tune Mistral-small. (B) In-domain and out-of-domain macro-*F*_1_ performance of all fine-tuned models. Solid bars show performance on the in-domain CSJDM test set, while striped bars show out-of-domain performance on the HOMO test set. Models shown in the left panel were fine-tuned using clinician-annotated training documents from CSJDM, whereas Mistral-small-psych (red, right panel) was fine-tuned using synthetic data only. For cross-site comparisons, results are restricted to concepts with at least 3 annotated instances in both test sets (n=97 concepts). Error bars represent 1 SD in *F*_1_-scores across evaluated concepts. CSJDM: Clínica San Juan de Dios Manizales; EHR: electronic health record; HOMO: Hospital Mental de Antioquia; LLM: large language model; QC: quality control; tNLP: traditional natural language processing.

### Cross-Testing

To assess their generalizability to other settings, all fine-tuned models, which were trained on the CSJDM training data or on synthetic data derived from CSJDM annotations, were also evaluated on the HOMO test set (n=309 documents; Figure 4B and Table 4). Moreover, the concept count distribution for both test sets is shown in Figure S7A in Multimedia Appendix 2. Though all LLMs showed a significant improvement from their zero-shot performance on the HOMO test set (Table S7 in Multimedia Appendix 1), the fine-tuned LLMs exhibited varying degrees of out-of-domain performance, with the larger models generalizing better than the smaller ones (Spearman ρ=–0.91; *P*<.001; Table 4). Fine-tuned Mixtral-8x7B showed the best generalizability, with a loss of only 0.02 (2.5%) in macro-*F*_1_, while Qwen2.5-7B had the greatest loss in performance (–0.16 points in macro-*F*_1_, or 19.75%), stemming mostly from a large drop in recall in the cross-evaluation (Table S8 in Multimedia Appendix 1).

Mistral-small-psych showed moderate out-of-domain performance, surpassing the macro-*F*_1_ performance of 3 of the LLMs fine-tuned on real data (Llama3-8B, Qwen2.5-7B, and Mistral-7B), and similar (within 0.05 range in macro-*F*_1_) to that of 4 LLMs fine-tuned on real data (Mistral-nemo, Qwen2.5-14B, Llama3-70B, and Aya-8B; Figure 4B).

**Table 4:** In-domain and out-of-domain performances of fine-tuned generative language models for psychiatric concept detection in Spanish electronic health records from Colombia^a^.

Model	In-domain testing (CSJDM test set)					Out-of-domain testing (HOMO test set)			
	Precision, mean (SD)	Recall, mean (SD)	Macro-*F*_1_, mean (SD)	Micro-*F*_1_	Precision, mean (SD)		Recall, mean (SD)	Macro-*F*_1_, mean (SD)	Micro-*F*_1_
Llama3-70B_finetuned	0.9 (0.14)	0.76 (0.19)	0.81 (0.16)	0.89	0.84 (0.21)		0.74 (0.23)	0.77 (0.21)	0.86
Llama3-8B_finetuned	0.9 (0.17)	0.79 (0.21)	0.83 (0.18)	0.90	0.85 (0.24)		0.64 (0.26)	0.70 (0.24)	0.81
Qwen2.5-14B_finetuned	0.89 (0.16)	0.8 (0.23)	0.82 (0.19)	0.91	0.87 (0.18)		0.74 (0.23)	0.77 (0.20)	0.86
Qwen2.5-32B_finetuned	0.89 (0.14)	0.86 (0.18)	0.87 (0.15)	0.93	0.86 (0.17)		0.76 (0.22)	0.79 (0.19)	0.87
Qwen2.5-7B_finetuned	0.89 (0.18)	0.76 (0.22)	0.81 (0.20)	0.88	0.82 (0.24)		0.58 (0.24)	0.65 (0.23)	0.76
Aya-35B_finetuned	0.9 (0.12)	0.85 (0.16)	0.86 (0.13)	0.92	0.87 (0.13)		0.81 (0.18)	0.83 (0.14)	0.88
Aya-8B_finetuned	0.88 (0.14)	0.82 (0.19)	0.83 (0.16)	0.91	0.81 (0.22)		0.72 (0.22)	0.74 (0.20)	0.84
Mistral-7B_finetuned	0.83 (0.21)	0.75 (0.24)	0.77 (0.22)	0.88	0.77 (0.27)		0.57 (0.27)	0.63 (0.26)	0.77
Mistral-nemo_finetuned	0.91 (0.14)	0.83 (0.19)	0.85 (0.16)	0.92	0.87 (0.19)		0.72 (0.23)	0.77 (0.20)	0.85
Mistral-small_finetuned	0.9 (0.15)	0.85 (0.20)	0.86 (0.16)	0.92	0.85 (0.23)		0.79 (0.26)	0.81 (0.23)	0.89
Mixtral-8x7B_finetuned	0.88 (0.15)	0.81 (0.20)	0.83 (0.17)	0.91	0.86 (0.14)		0.80 (0.18)	0.81 (0.14)	0.87
Mistral-small-psych	0.84 (0.17)	0.79 (0.16)	0.81 (0.15)	0.85	0.83 (0.19)		0.68 (0.20)	0.73 (0.17)	0.79

^a^Mean precision, recall, macro-*F*_1_-score, and micro-*F*_1_-score for fine-tuned large language models evaluated on the in-domain Clínica San Juan de Dios Manizales (CSJDM) test set (n=358 documents) and the out-of-domain test set from Hospital Mental de Antioquia (HOMO; n=309 documents). All models except Mistral-small-psych were fine-tuned using clinician-annotated training documents from CSJDM; Mistral-small-psych (bottom) was fine-tuned using synthetic data containing CSJDM concept annotations. Both test sets were annotated using the same framework by the same clinical annotation team. Results are restricted to psychiatric concepts with at least 3 annotated instances in both test sets (n=97 concepts). Precision, recall, and macro-*F*_1_ are reported as mean (SD) across phenotype-specific *F*_1_-scores. Micro-*F*_1_ is a single aggregate score calculated by pooling true positives, false positives, and false negatives across all evaluated phenotypes and therefore is not accompanied by an across-phenotype SD.

### Synthetic Training Set Size Sensitivity Analysis

To assess the sensitivity of Mistral-small-psych’s fine-tuning process to the synthetic training data size—and the potential gains from a larger synthetic training set—we created random subsets of increasing sizes in 250 document increments. Three random subsets of each size were sampled from the original synthetic training data, and Mistral-small was fine-tuned on each training set and evaluated on the CSJDM test set. We used 3 replicates at each training-set size to estimate variability due to random sampling and to determine whether performance was robust to the specific synthetic documents selected, while limiting the computational cost of repeated LLM fine-tuning.

Performance converged quickly in all aspects (mean *F*_1_, precision, and recall; Figure S9 in Multimedia Appendix 2). Strikingly, a training set of only 750 documents was sufficient to reach mean recall and *F*_1_-scores of 0.77 and 0.78, nearly matching the performance achieved using the full synthetic training set. Moreover, the random samples of each size category yielded highly similar results (performance within 0.01 of the mean *F*_1_ values for each subset size), suggesting the training procedure’s robustness to varying selections of synthetic training documents.

To assess whether these results could be artifacts attributed to prevalence distortions in random subsamplings, we conducted a concept-level prevalence stability analysis (Table S11 in Multimedia Appendix 1). After excluding ultra-rare concepts (<3 instances in either the test set or training set), we compared concept distribution similarities in the random subsets and full training set. Correlations between concept count vectors remained consistently high across subsets, with little variation between random draws (Spearman ρ ranging from 0.94 to 0.95 for 250-document subsets and >0.99 for larger subsets). Distributional similarity metrics further demonstrated minimal prevalence drift, with Jensen-Shannon divergence values <0.02 across all subsets and small Hellinger distances between normalized prevalence vectors. Additionally, the number of concepts absent from a given subset was low. Together, these results indicate that the observed performance saturation is unlikely to be driven by random sampling-induced artifacts or prevalence drifts, instead reflecting genuine robustness of the fine-tuning process to synthetic training set size and random sampling.

### Error Analysis

The full concept-level error statistics (true positive, false positive, false negative, and true negative) for zero-shot, few-shot, and fine-tuned LLMs are shown in full in Tables S2-S4 in Multimedia Appendix 1, respectively. As previously noted, most LLMs exhibited poor performance with zero-shot prompting, largely due to high false positive rates. Few-shot prompting mitigated (but did not fully solve) this issue, in most cases.

For the fine-tuned models, we observed moderate to strong correlations between performance and frequency in the training data (Spearman ρ=0.32-0.55; *P*<.001; Figure S6D in Multimedia Appendix 2), as well as interannotator agreement (Cohen κ, Spearman ρ=0.46-0.61; *P*<.001, Figure S6E in Multimedia Appendix 2), in line with previous findings [33].

The concepts with the greatest improvement in *F*_1_ across LLMs following fine-tuning compared with zero-shot prompting of base models were “altered emotional resonance,” “hypothymia,” “altered prospection,” and “hypomanic symptoms” (all with increases of over +0.7 in average *F*_1_; Table S4 in Multimedia Appendix 1). Interestingly, most of these concepts had very low training set frequency (of up to 15 annotated instances in over 1400 documents) but highly repetitive test set annotations deviating little from the concept name (Table S9 in Multimedia Appendix 1), suggesting that low training-set frequency does not necessarily preclude high performance when annotations are lexically consistent and closely aligned with the concept definition.

In contrast, some broader concepts (“poor response to psychotropic medications,” “other addictions,” “disrobing,” and “obsessions”) saw little improvement from their zero-shot performance after fine-tuning, mostly due to poor recall across models. For the most part, these concepts exhibited lower training and test set frequency (<20 instances in the training set) and diverse annotations in the test set, highlighting the difficulty all LLMs faced to extrapolate and capture these instances based on the limited training data, in light of their lexical variability. Moreover, one concept (“TECAR”) showed a decrease in *F*_1_ after fine-tuning relative to the base models’ zero-shot performance on average across all models (mean decrease=–0.3 *F*_1_, SD 0.43), driven primarily by reduced recall. Notably, this occurred even though all test-set annotations of “TECAR” contained the terms “tecar” or “tec-ar,” both of which were represented in the training data (Table S9 in Multimedia Appendix 1). Along similar lines, we found weak to moderate correlations (Spearman ρ=0.25-0.44; *P*<.05 for all LLMs) between fine-tuned LLM performance (*F*_1_) and a proxy measure of concept lexical consistency, defined as the ratio of each concept’s total support to the number of distinct patterns specified in the tNLP method based on annotated expressions in the CSJDM training set (Figure S6F in Multimedia Appendix 2). These associations further suggest that concepts characterized by greater lexical variability and ambiguity may remain more challenging for models to learn and detect. Together, these findings suggest that concept-level performance was not determined by training frequency alone, but rather by the interaction between training-set support and annotation consistency or semantic breadth.

While many concepts showed consistent gains or drops in performance across fine-tuned models, some showed low performance specific to one model (eg, “poor adherence” for Qwen2.5-7B or “fatigue” for Llama3-70B; Figure 3 and Table S4 in Multimedia Appendix 1), indicating model-specific limitations in learning these concepts, rather than an issue stemming from inherent characteristics of the training or testing data.

For Mistral-small-psych, lower performance typically stemmed from low recall. We also observed a tendency to highlight spans with new concepts that did not appear in the training data, such as medication labels and categories (“antidepressants”), psychomotor and behavioral concepts (“collaboration”), and somatic concepts (“hypertension”; Table S9 in Multimedia Appendix 1). The number of predicted labels for these concepts fluctuated with training duration, peaking at 9 epochs and decreasing as training continued further (Figure S10 in Multimedia Appendix 2).

Finally, as a descriptive assessment of whether Mistral-small-psych simply replicated tNLP behavior, we compared concept-level recall across overlapping concepts. We found that Mistral-small-psych showed strictly higher recall than tNLP for 30 of 88 overlapping concepts, including “poor personal hygiene,” “weight loss,” “poor concentration,” and “perseveration.” Several differences reflected lexically variable mentions captured by the LLM but not by the pattern-based system (eg, “bathed the other day”), illustrating that the synthetic-trained model was not merely reproducing tNLP pattern behavior.

### Inference Runtime, Electricity Consumption, and Cost

Two important limitations of generative AI are cost and carbon footprint. Larger models had longer runtimes and greater electricity consumption (Table S10 in Multimedia Appendix 1; reported times include writing the generated outputs to an output file, but exclude model loading time and JSON parsing), with Aya-35B and Llama3-70B having the longest runtime (85.62 and 81.11 seconds to process 100 documents, respectively). Llama3-70B also had the highest electricity consumption (12.2 watt-hours), nearly 7-fold that of its smaller counterpart Llama3-8B.

## Discussion

### Principal Findings

In this study, we evaluated the ability of 11 state-of-the-art decoder-based LLMs to detect a wide range of psychiatric symptom-level concepts in real EHR notes, assessing both “out-of-the-box” and fine-tuned performance, including external validation. Our contributions are 2-fold: first, to the best of our knowledge, this is the largest study on the ability of generative AI to extract psychiatric symptoms from EHRs, both in terms of the number of models evaluated and the number of concepts targeted. These findings are important because psychiatric concept detection encompasses several NLP challenges: sparse labels, variable and occasionally ambiguous symptom expression, reliance on subjective accounts, regional or specific jargon (eg, for substance-related terms), and the need to distinguish clinically meaningful concepts from surrounding contextual information.

Second, we focus on Spanish clinical text, aiming to help close the gap in clinical NLP research and applications in non-English settings. With nearly 500 million native Spanish speakers worldwide and widespread clinical use across multiple countries, the development, evaluation, and publication of such tools is important for ensuring precision psychiatry and clinical advancements in globally diverse populations.

We created an open-source fine-tuned generative LLM, Mistral-small-psych, for the extensive extraction of psychiatric concepts from Spanish-language clinical text and EHR documents. This privacy-preserving open-source LLM is easy to deploy and use and can be further fine-tuned with additional annotated data for further adjustment to local documentation practices. Furthermore, we use a fully synthetic training framework based on injecting expert-annotated concept spans into AI-generated sentences, incorporating somatic (nonpsychiatric) clinical sentences, and augmenting recall via weak-labeling with a validated tNLP system. We show that an LLM fine-tuned solely on this synthetic training set achieves performance and cross-site generalizability comparable to those of models trained on the full real EHR corpus. In practice, this LLM can be safely applied as is in clinical settings for the task of fine-grained automatic symptom detection from medical records in Spanish or further tuned on local annotated corpora. Moreover, with appropriate postprocessing, context resolution, longitudinal aggregation, and local validation, these extracted concepts could serve as inputs for downstream applications such as medical record summarization, symptom tracking, and research beyond diagnostic coding.

### Model Performance and Comparison to Prior Work

Our results demonstrate that zero- and few-shot model performance was generally limited due to a tendency to overpredict positives. Nevertheless, few-shot prompting yielded some improvement in both recall and precision over zero-shot performance in most cases. In addition, larger models did not necessarily yield better results overall. The limited out-of-the-box performance on this task could be attributed in part to the following 2 factors: first, the specificity of the task itself, as psychiatric notes contain domain-specific language and terms, as well as nuanced symptomatology; second, the relatively small fraction of Spanish-language data in the LLMs’ pretraining corpora (for instance, for Llama3 models, less than 10% of the total pretraining data corresponded to all non-English language tokens, combined [34,45]). Therefore, similar analyses in English EHRs may yield significantly better zero- and few-shot performance, given the greater representation of English in LLM training data. However, English psychiatric EHRs would still likely require some task-specific adaptation, because the core challenges of psychiatric concept detection, including local documentation practices, sparse labels, and ambiguous or context-dependent mentions, are not unique to Spanish.

Fine-tuning led to dramatic performance gains in all models, with the top-performing models (Aya-35B, Qwen2.5-32B, and Mistral-small) achieving a macro-*F*_1_-score of 0.86; moreover, several fine-tuned models achieved higher macro-*F*_1_-scores compared to the tNLP method developed from the same data. In line with the literature [46,47], these findings showcase the importance of task-specific model adaptation in enabling LLMs to perform effectively in clinical phenotyping.

Fine-tuned LLMs generally performed better on concepts that were more common in the training data, but this relationship was not uniform and should not be interpreted as a simple data-volume effect. Concept-level performance appeared to reflect the interaction of training-set frequency with other factors, including lexical variability and semantic breadth, and concept complexity. Other errors were more model-specific, such as Qwen2.5-7B’s lower performance on “poor adherence,” suggesting that some failure modes may also reflect model-specific limitations rather than concept-level difficulty alone. Consistent with these observations, the associations between model performance, interannotator agreement, and lexical variability suggest that ambiguity, expert-level interpretive difficulty, and expression diversity remain important sources of error in psychiatric concept detection and ongoing challenges for clinical NLP approaches more broadly.

Additionally, we emphasize that Mistral-small-psych was not exposed to the tNLP patterns during training. Moreover, our results suggest that its behavior differed from the pattern-based system: Mistral-small-psych achieved higher recall than tNLP for dozens of concepts, suggesting that the LLM captures lexically variable mentions not covered by the fixed patterns and did not simply perform knowledge distillation. This highlights a general advantage of generative LLMs for concept extraction: while tNLP systems can be highly precise, their recall may be limited by their rigidity. In contrast, an LLM may generalize to alternative phrasings. Nevertheless, because the synthetic training corpus was weakly labeled by the tNLP system, Mistral-small-psych may have inherited some of the rule-based system’s biases or blind spots.

Finally, cross-evaluation of the fine-tuned LLMs demonstrated their overall generalizability, though the degree of out-of-domain performance loss varied by model and concept, mostly driven by changes in recall. Larger LLMs generalized better to the new setting, in line with previous findings on LLM generalization [48,49].

### Practical Considerations

Benchmarking results revealed substantial variability in runtime and cost across the evaluated LLMs, and these differences would compound when applying these models to large repositories of clinical notes. Hence, we prioritized accessibility [23] in the creation of Mistral-small-psych by selecting Mistral-small, which yielded the smallest top-performing model upon fine-tuning on real data, as our base model. Despite being trained solely on AI-generated content, Mistral-small-psych exhibited high precision—suggesting a low rate of false positives against the reference annotations—and small performance drops in the external validation.

Training data size sensitivity analysis showed that fine-tuning on approximately 60% of the synthetic training data leads to nearly equal performance as fine-tuning on the full dataset, suggesting diminishing benefit from additional synthetic training data and highlighting the effectiveness of the fine-tuning process, even with relatively small (<1000 documents) fine-tuning corpora, and demonstrating the efficiency and scalability of the synthetic data–based approach.

In addition, in rare instances, base models did not engage in response to notes containing highly sensitive psychiatric content, occasionally producing safety-oriented or cautionary responses rather than the requested structured output, which were parsed as negative responses; this highlights a potential limitation of applying general-purpose instruction-tuned models to psychiatric EHR text without task-specific adaptation.

### Limitations and Future Directions

This study has limitations and highlights important directions for future work.

First, the performance of fine-tuned LLMs depends on the annotated corpus used for training and evaluation. These models may therefore be sensitive to data drift, changes in documentation practices, varying patient populations, or institutional workflows. We partially addressed this limitation by cross-evaluation on an external test set; however, like any clinical machine learning system, they should be periodically re-evaluated and updated to ensure reliability.

Second, although fine-tuned models were trained to output both detected concepts and their associated text spans, this provides only limited interpretability. Span outputs make predictions easier to inspect and can facilitate validation and error analyses, but they do not fully explain the models’ decision-making procedure or confirm the clinical accuracy of the extracted concepts. Future work could include more in-depth evaluations of model predictions using counterfactual alterations and expert clinician review of whether the returned spans provide sufficient evidence for each prediction.

Third, this study was designed to evaluate decoder-only generative LLMs and did not include encoder-only models, such as BERT (Bidirectional Encoder Representations from Transformers). These architectures may offer comparable performance to LLMs, while being smaller and more computationally efficient. While we included comparisons with a tNLP baseline and evaluated multiple LLMs of various families and sizes, future work should systematically compare generative LLMs with different encoder-based models to better assess the trade-offs between generalization capacity, cost and efficiency, and concept-specific performance.

Fourth, while we assessed model transportability through cross-site evaluation using an external test set from an independent hospital, both datasets came from psychiatric hospitals in Colombia. Clinical Spanish can vary across countries, including differences in vocabulary, abbreviations, colloquial symptom descriptions, and documentation practices. As a result, the observed generalizability may not extend to other Spanish-speaking settings. In addition, the 2 test sets differed in the clinical note fields included, as sections selected for annotation at each institution were chosen to prioritize fields with the highest concentration of symptoms. This difference may have itself influenced cross-site performance. Future work should evaluate these models across additional Spanish-speaking countries, while also assessing the impact of note composition on model performance.

Fifth, this study evaluated explicit psychiatric concept mention detection rather than assertion status–resolved patient-level phenotyping. This design allowed us to benchmark a challenging first-stage extraction task: detecting over 100 fine-grained psychiatric concepts from Spanish clinical text, a setting with limited annotated resources, and few prior benchmarks. Requiring models to simultaneously determine whether each mention was current, negated, historical, hypothetical, uncertain, or described for another person would add an additional layer of complexity requiring separate validation and benchmarking, and unprocessed model outputs should not be interpreted as current patient symptoms. To partially address this limitation, the models fine-tuned on CSJDM data were trained to also identify and output context cues, including negation, temporality, family history, hypotheticals, and uncertainty. Although context-cue prediction was not the primary benchmark evaluated in this study, these outputs provide a basis for downstream postprocessing to distinguish current patient symptoms from historical, nonasserted, or nonpatient mentions. Relatedly, while fine-tuning and testing prompts were consistent for this task, the synthetic sentence generation prompt did not explicitly sample negated, historical, hypothetical, uncertain, or nonpatient mentions in a controlled way, which may have limited Mistral-small-psych’s robustness to some contexts. Future work should explicitly evaluate context resolution to convert extracted concepts into clinically actionable patient-level phenotypes and explicitly introduce balanced assertion-status variants into synthetic data generation.

Sixth, although the fine-tuned LLMs were evaluated against a predefined psychiatric concept ontology, outputs were generated using unconstrained decoding. Consequently, models occasionally produced labels outside the target concept list, such as medication classes. These predictions were excluded from our evaluation, and while they could be potentially useful for downstream applications, they highlight a limitation of generative LLMs in clinical settings, where unconstrained outputs could produce irrelevant outputs if not postprocessed appropriately. Future work could explore ontology-constrained decoding to reduce out-of-ontology generations.

Finally, we used 4-bit quantized LLMs, which is common practice in LLM use, to make large-scale applications computationally feasible. This quantization may have resulted in reduced performance relative to full precision. Moreover, this limitation may be particularly relevant for non-English text, seeing that quantization may disproportionately affect linguistic representations less prevalent in the models’ original pretraining data, including Spanish tokens and psychiatric terminology. Consequently, the performances reported here may underestimate the optimal achievable performances of these LLMs under full precision. Future work could directly compare full-precision versions of the same models to better estimate the magnitude of this effect.

### Conclusions

This study presents the large-scale evaluation and fine-tuning of multiple generative LLMs for the extraction of over 100 transdiagnostic psychiatric symptoms from Spanish clinical text, showing that several fine-tuned models achieved strong performance and outperformed a previously developed tNLP method for many concepts. In addition, we developed an open-source generative LLM fine-tuned using synthetic clinical text, which achieved performance comparable to models trained on real EHR data while remaining fully shareable. Finally, cross-site evaluation demonstrated that most fine-tuned models retained good transportability with relatively modest performance declines in external settings. Together, these findings support the feasibility of developing shareable, locally adaptable clinical NLP tools for psychiatric concept extraction in underresourced language settings.

## Appendix

Multimedia Appendix 1 Supplementary Tables ST1-ST11 providing detailed information and results for the evaluated models, psychiatric phenotypes, and experimental settings.

Multimedia Appendix 2 Supplementary notes and figures (figures S1-S10) providing additional methodological details and analyses for the LLM inference, fine-tuning, and evaluation experiments.

## Abbreviations

AWS: Amazon Web Services
BERT: Bidirectional Encoder Representations from Transformers
CSJDM: Clínica San Juan de Dios Manizales
EHR: electronic health record
GPU: graphics processing unit
HOMO: Hospital Mental de Antioquia
LLM: large language model
LoRA: low-rank adaptation
NLP: natural language processing
TECAR: terapia electroconvulsiva con anestesia y relajación
tNLP: traditional natural language processing
VRAM: video random-access memory

## Data Availability

The full annotated dataset used to evaluate and fine-tune the large language models (LLMs) is not publicly available because it comprises clinical notes containing patient data and is subject to privacy, confidentiality, and institutional data use restrictions. The specific text spans annotated for the study were previously published by De La Hoz et al [33]. The synthetic dataset used to create Mistral-small-psych and the code used for inference, fine-tuning, and evaluation are available in the LLMs for Psychiatric Phenotyping GitHub repository [50]. The open-source Mistral-small-psych model is publicly available through Hugging Face [51]. The complete set of patterns developed for the traditional natural language processing method is publicly available in the Spanish Psych Phenotyping GitHub repository [52].
