# Supplementary material for: Generative Large Language Models for Psychiatric Concept Detection in Spanish-Language Electronic Health Records: Development and Evaluation Study
Source: J Med Internet Res. 2026 Oct 26;28:e93328. doi: 10.2196/93328 (PMC13749023; doi:10.2196/93328)
Supplement: Supplementary file 1 [file jmir_v28i1e93328_app2.docx]

**Figure S1. Relationship between model size and psychiatric concept extraction performance for zero-shot, few-shot, and fine-tuned generative large language models.**

Model size (measured in billions of parameters) is shown for 11 open-source generative large language models evaluated on the Clínica San Juan de Dios Manizales test set (n=358). Panels show performance metrics (mean F1 [1], precision [2] and recall [3]) using zero-shot prompting (A), few-shot prompting with up to five annotated examples of the target concept included in the prompt (B), and after fine-tuning on clinician-annotated Clínica San Juan de Dios Manizales training documents (C).

**Figure S2. Zero-shot concept-level performance of generative large language models for psychiatric concept extraction from Spanish electronic health record documents.**

(A) Per-concept F1 scores for each of the 11 generative large language models evaluated using zero-shot prompting on the Clínica San Juan de Dios Manizales test set (n=358 documents). Concepts are ordered by their frequency in the Clínica San Juan de Dios Manizales training set.

(B) Zero-shot per-concept F1 scores for the generative large language models (colored circles) compared with the pattern-based traditional natural language processing method (black crosses) on the Clínica San Juan de Dios Manizales test set. The traditional natural language processing method’s performance is shown only for the 88 concepts for which it was developed and evaluated, among the 109 concepts evaluated for the large language models. Concepts are ordered by the traditional natural language processing method’s performance.

**Figure S3. Mean performance of zero-shot, few-shot, and fine-tuned generative large language models compared with a traditional natural language processing method for psychiatric concept extraction from Spanish electronic health record documents.**

Mean F1 scores (macro-F1) are shown for 11 generative large language models evaluated on the Clínica San Juan de Dios Manizales test set (n=358 electronic health record documents) using zero-shot prompting, few-shot prompting, and fine-tuning on a training set of clinician-annotated documents from Clínica San Juan de Dios Manizales. The pattern-based traditional natural language processing method (shown in black at right) was developed from the same training set, and is included for comparison. Results are restricted to the 88 psychiatric concepts evaluated by both the large language models and the traditional natural language processing method. Error bars indicate one standard deviation across evaluated concepts.

**Figure S4. Few-shot concept-level performance of generative large language models for psychiatric concept extraction from Spanish electronic health record documents.**

(A) Per-concept F1 scores for each of the 11 generative large language models evaluated using few-shot prompting on the Clínica San Juan de Dios Manizales test set (n=358 documents). For each concept, up to five examples of annotated instances from the training set were provided as part of prompting. Concepts are ordered by their frequency in the Clínica San Juan de Dios Manizales training set.

(B) Few-shot per-concept F1 scores for the generative large language models (colored circles) compared with the pattern-based traditional natural language processing method (black crosses) on the Clínica San Juan de Dios Manizales test set. The traditional natural language processing method’s performance is shown only for the 88 concepts for which it was developed and evaluated, among the 109 concepts evaluated for the large language models. Concepts are ordered by the traditional natural language processing method’s performance.

**Figure S5. Concept-level performance of generative large language models fine-tuned on clinician-annotated Spanish electronic health record documents from Colombia.**

(A) Per-concept F1 scores for each of the 11 generative large language models fine-tuned on clinician-annotated training documents from Clínica San Juan de Dios Manizales (n=1,477 training documents) and evaluated on the Clínica San Juan de Dios Manizales test set (n=358 documents). Concepts are ordered by their frequency in the training set.

(B) Per-concept F1 scores for the fine-tuned generative large language models (colored circles) are compared with the pattern-based traditional natural language processing method (black crosses) developed from the same training set. All models were evaluated on the Clínica San Juan de Dios Manizales test set. The traditional natural language processing method is shown only for the 88 concepts for which it was developed and evaluated, among the 109 concepts evaluated for the large language models.

**f**

**Figure S6. Concept-level performance metrics of fine-tuned generative large language models in relation to concept frequency, inter-annotator agreement, and lexical variability.**

Per-concept recall (A), precision (B), and F1 score (C) are shown for generative large language models fine-tuned on clinician-annotated training documents from Clínica San Juan de Dios Manizales (n=1,477 training documents) and evaluated on the Clínica San Juan de Dios Manizales test set (n=358 documents), plotted against log-transformed concept frequency in the training set. Per-concept F1 scores are also plotted against concept frequency in the test set (D), inter-annotator agreement in the training set measured using Cohen’s kappa (E), and a proxy measure of lexical variability calculated as the ratio of total concept frequency to the number of patterns developed from annotated expressions in the Clínica San Juan de Dios Manizales training data (F). Spearman correlation coefficients and P values are reported for the associations.

**a**

**b**

**Figure S7. Psychiatric concept frequencies in the test sets, as well as in the synthetic training set.**

(A) Frequency of each psychiatric concept in the Clínica San Juan de Dios Manizales and Hospital Mental de Antioquia test sets (top and bottom panels), used for in-domain and out-of-domain model evaluation, respectively. Concepts are ordered by their frequency in the Clínica San Juan de Dios Manizales test set.

(B) Frequency of each psychiatric concept in the synthetic training set used to fine-tune Mistral-small-psych (n=1,223 synthetic documents).

**Figure S8.** Concept-level F1 performance of Mistral-small-psych (red), alongside the baseline zero-shot performance of the 11 generative LLMs evaluated on the Clínica San Juan de Dios Manizales test set. The best baseline LLM performance for each concept is highlighted in black rhombi. Concepts are ordered by the difference between Mistral-small-psych’s F1-score and the F1-score achieved by the top performing baseline model for that concept. Mistral-small-psych strictly outperformed the best-performing baseline LLM in 75 of the 109 concepts evaluated.

**Figure S9. Synthetic training set size sensitivity analysis for Mistral-small-psych.**

Saturation curves show Mistral-small-psych’s performance on the Clínica San Juan de Dios Manizales test set (n=358 documents) as a function of synthetic training set size. Panels show mean F1 score (A), recall (B), and precision (C) after fine-tuning on randomly sampled subsets of the synthetic training documents. Training set sizes included 0 documents, representing the zero-shot performance of the base Mistral-small model before fine-tuning; 250, 500, 750, and 1000 synthetic documents; and the full synthetic training set (n=1,223 documents). For intermediate subset sizes, points represent the mean across three independent random samples, and error bars indicate the minimum and maximum performance values observed across replicates.

**Figure S10. Incidental concept labeling by Mistral-small-psych across synthetic-data fine-tuning epochs.**

This figure shows the number of unique incidental psychiatric concepts (not included as target labels in the original synthetic training examples) flagged by Mistral-small-psych across training epochs on the synthetic data. Higher counts indicate recovery of additional concept labels incidentally predicted to be present in the test set.

**Notes:**

**Note 1: Synthetic Dataset Creation**

To create the initial pool of synthetic sentences, we utilized Llama3-70B, which was found to produce the best results among all LLMs tested in this study through an empirical comparison using different prompting strategies.

For each concept, we prompted Llama3-70B, providing CSJDM training set annotations (hereafter, “literals”), as published in ref [1]. In addition, to emulate the real CSJDM EHR training data, we prompted the LLM to produce sentences with no mental-health-related symptoms, but containing somatic symptoms, laboratory test results, and medical history (hence “somatic/other sentences”). The following prompts were used to generate synthetic sentences for concept *P,* and negative sentences, respectively:

| Prompt | Temperature |
| --- | --- |
| “You are a Spanish-speaking clinician. Here are a few examples of mentions of the concept <P>: <annotation_example_list>. Imagine you are a Spanish-speaking clinician. Please generate at least 100 sentences from your clinical notes in Spanish about various patients with instances of the symptom <P>, each sentence containing one of these examples. You must generate at least one sentence for every example I provided. Use the examples exactly as I provided them (but without quotes). All sentences must be in Spanish, with a new line for each sentence. After each sentence, include a pipe ('\|') and then the example mention that you included in the note, exactly as I provided it.” | 0.8 |
| “Imagine you are a Spanish-speaking clinician. Please generate at least 40 [100] sentences from your clinical notes in Spanish about various patients, that do not contain any mentions of psychiatric symptoms. Sentences can contain information about the patients' age, residence, occupation, living situation, treatment plans and clinical history. Sentences should also contain information on lab test results, medications and prescriptions (name of medications and dosages), past medication history, quotes from the patient, and general somatic health conditions, but no psychiatric or mental health-related conditions." | 0.99 |

The LLM was served via Ollama, on an AWS instance with 4 A10G Tensor Core GPUs with a total of 96 GB of VRAM. Llama3-70B was prompted to generate sentences in batches of 40-100 sentences. Then, a quality control step was applied, in which numberings were removed and sentences not containing their assigned annotation literals were filtered out. The somatic/other clinical sentences were also reviewed for coherence, plausibility, and absence of overt psychiatric symptom mentions. These sentences were included to approximate the nonpsychiatric clinical and demographic content commonly present in real EHR notes, rather than to serve as formal negative examples for a binary classifier. The higher generation temperature was used to reduce repetition across batches generated from similar prompts. After assembly into synthetic documents, the tNLP weak-labeling step was applied to the synthetic corpus, allowing incidental psychiatric concept mentions appearing in generated text to be labeled rather than treated as absent.

Next, we randomly sampled sentences for each concept, prioritizing the inclusion of all distinct literals before sampling additional sentences until reaching the frequency of the concept in the original CSJDM training data, if necessary. This process yielded a total of 3,440 sentences, corresponding to 119 concepts.

In addition, 100 sentences were randomly converted to uppercase, imitating the style in the real CSJDM text. Then, we randomly sampled 300 somatic/other sentences, for a total of 3,740 sentences. All sentences were shuffled and assembled into groups of 1-6 sentences, for a total of 1,223 synthetic documents.

Since the generated synthetic sentences were intended to emulate free text written by clinicians about psychiatric patients, they often contained additional descriptions of psychiatric concepts, other than the ones explicitly prompted for. To also label these additional symptoms incidentally appearing in the synthetic dataset and create a more accurately labeled training dataset, we applied the tNLP method to the synthetic documents as a weak-labeling approach.

Finally, the dataset was converted into alpaca format, with the input being the documents and the output being the labeled spans and their corresponding concept labels**.**

**Note 2: Zero-Shot Inference**

The following prompt was used to query each LLM for the detection of concept *P* in document *D*:

“The following is a document in Spanish for a controlled and safe analysis task. Respond '1' if this text contains an explicit mention or instance of the concept: <P>, and '0' otherwise. Respond '1' even if the symptom appears in a negated, hypothetical, or historical context, or used to describe a different person. Do not assume the presence of the symptom if it does not explicitly appear in the note in a mention or example. Reply just '1' or '0', nothing more. The note is: <D>”

No additional information was provided to the models, and no context was carried across queries. Responses given in the wrong format (most often general descriptions and reiterations of the document or statement that the document does not contain an explicit mention of the document, e.g.: “The note does not contain an explicit mention or instance of the concept ‘Abulia’”) were classified as negative responses. These non-binary responses converted to negative predictions were reviewed and were found to primarily consist of safety-oriented refusals to engage with the instruction, summarization of the themes expressed in the note followed by action recommendations for the experiencer, or explanatory negative answers rather than positive assertions.

For higher reproducibility and output stability, all evaluations were done under temperature=0.

**Note 3: Few-Shot Inference**

For few-shot prompting, up to five distinct annotations of each concept were provided to the LLMs as examples in the prompt, along with the document. All examples were selected from the CSJDM training set. Selecting the most common annotated spans in the training set often results in highly similar spans (for example, for the concept “psychotic symptoms”, the most common annotations were “SÍNTOMAS PSICÓTICOS”, “sintomas psicóticos”, and “SINTOMAS PSICOTICOS” – all highly similar spans, introducing little variation and no alternative phrasing). Therefore, to balance representativeness and diversity, concept annotation examples were selected using the following strategy: first, we ranked all annotated instances in the training set by frequency, and then randomly selected five examples from the top ten most common different annotations. If no more than ten or five different annotations were annotated for the concept, we selected all instances to randomly sample from, or all different annotated instances to construct the few-shot prompt, respectively. This ensured that we would include common annotations as few-shot examples, providing power to the prompt, while also introducing some diversity. For each concept, the same examples were provided when prompting for each test set document.

The following prompt was used to query each LLM for the detection of concept *P* in document *D,* given annotated example list *E*:

"I will provide a document in Spanish for a controlled and safe analysis task.

Your task is to respond '1' if this note contains an explicit mention or instance of the concept: <P>, and '0' otherwise. Respond '1' even if the symptom appears in a negated, hypothetical, or historical context, or is described for a different person. Do not assume the presence of the symptom if it does not explicitly appear in the note in a mention or example. Reply just '1' or '0', nothing more. Here are a few examples of mentions of the concept <P>: <E>. And here is the document: <D>"

As in zero-shot prompting, no context was carried across queries, and responses not addressing the task (reiterations of the document’s content or explanations of why the document did not contain a given concept) were classified as negative responses.

**Note 4: Fine-Tuning on Real CSJDM Data**

Models were fine-tuned using a training set consisting of 1,477 documents and a validation set of 165 documents, used to fine-tune hyperparameters. Fine-tuning was carried out on g5.2xlarge (all Mistral and Qwen2.5 models, Llama3-8B, and Aya-8B) or g5.12xlarge (Llama3-70B, Aya-35B and Mixtral-8x7B) AWS instances, with 1/4 A10 NVIDIA Tensor Core GPUs and 24/96 GB of VRAM, respectively. Base models were downloaded in 4-bit quantization and fine-tuned using LoRA (with r=8, alpha=16 and dropout=0.05), targeting all model linear layers. Brain floating point 16-bit (bfloat16) precision was used. When utilizing multi-GPU instances, models were sharded across GPUs using Fully Sharded Data Parallel (FSDP). Unsloth was utilized to reduce memory consumption in single-GPU settings for all Mistral and Qwen2.5 models, as well as Llama3-8B. Axolotl was used for the fine-tuning of all other LLMs.

Training and validation data were converted to alpaca format, as follows:

| **Alpaca prompt** | Below is an instruction that describes a task, paired with an input that provides further context. Write a response that appropriately completes the request. |
| --- | --- |
| **Instruction** | ### Instruction:  I will provide a clinical note in Spanish. Identify a list of all symptoms from the input text, even if the symptom appears in a negated, hypothetical, or historical context, or described a different person. Format your answer as a list of the phenotypes separated by newline characters and do not generate random answers. Only output the list. |
| **Input** | ### Input:  <DOCUMENT_BODY> |
| **Output** | ### Response:  <PHENOTYPE_1_TEXT>\| <PHENOTYPE_1_LABEL> <PHENOTYPE_2_TEXT>\| <PHENOTYPE_2_LABEL> <PHENOTYPE_3_TEXT>\| <PHENOTYPE_3_LABEL>  …  END<\|im_end\|> |

Models were trained using mostly default parameters, for 1-4 epochs. For all models, we tested the following learning rates: 1e-3, 3e-4, 1e-4, 1e-5, as well as simulating a larger batch size using gradient accumulation steps=4.

The final training details for all models are shown below:

| **Model** | **Learning rate** | **Num epochs** | **Gradient accumulation steps** | **Training Time (Minutes)** | **Instance VRAM (GB)** |
| --- | --- | --- | --- | --- | --- |
| Llama3-8B | 3e-4 | 2 | 4 | 39 | 24 |
| Mistral-7B | 1e-5 | 3 | 1 | 81 | 24 |
| Mistral-nemo | 1e-4 | 2 | 1 | 52 | 24 |
| Mistral-small | 1e-4 | 2 | 4 | 98 | 24 |
| Qwen2.5-7B | 3e-4 | 2 | 4 | 36 | 24 |
| Qwen2.5-14B | 3e-4 | 2 | 4 | 92 | 24 |
| Qwen2.5-32B | 3e-4 | 2 | 4 | 116 | 24 |
| Aya-8B | 1e-3 | 2 | 4 | 46 | 24 |
| Aya-35B | 3e-4 | 4 | 4 | 178 | 96 |
| Llama3-70B | 3e-4 | 2 | 4 | 581 | 96 |
| Mixtral-8x7B | 3e-4 | 3 | 4 | 196 | 96 |

**Note 5: Fine-Tuning on Synthetic Data**

To create Mistral-small-psych, Mistral-small was fine-tuned for 9 epochs (after testing 1-14) on the synthetic dataset documents (n=1,223), on an AWS g5.2xlarge instance with one A10 NVIDIA Tensor Core GPU with 24 GB of VRAM. Documents were converted to the alpaca format for training (see **Note 4** for template). The base model was fine-tuned using a learning rate of 3e-4 and a larger batch size was simulated using a gradient accumulation step of 16. As with the fine-tuning on real data, the base LLM was downloaded in 4-bit quantization and fine-tuned using LoRA (r=8, alpha=16 and dropout=0.05) targeting all model linear layers and using Bf16. Unsloth was utilized to reduce memory consumption and training time. Total training time (for 9 epochs) was 4 hours.

**Note 6: Evaluation Metrics**

Performance at all levels (for both inference and fine-tuned models) was evaluated on the CSJDM test set against the clinicians’ annotations, using precision, recall, macro-F1, micro-F1, and balanced accuracy, defined as follows:

Let $k=1,\ldots,K$ index the evaluated concepts, and let $TP_{k}$, $FP_{k}$, $FN_{k}$, and $TN_{k}$ denote true positives, false positives, false negatives, and true negatives for concept $k$.
